# The Study on the hERG Blocker Prediction Using Chemical Fingerprint Analysis

**DOI:** 10.3390/molecules25112615

**Published:** 2020-06-04

**Authors:** Kwang-Eun Choi, Anand Balupuri, Nam Sook Kang

**Affiliations:** Graduate School of New Drug Discovery and Development, Chungnam National University, 99 Daehak-ro, Yuseong-gu, Daejeon 34134, Korea; hwendiv@naver.com (K.-E.C.); balupuri@cnu.ac.kr (A.B.)

**Keywords:** hERG toxicity, drug discovery, fingerprints, machine learning, deep learning

## Abstract

Human ether-a-go-go-related gene (hERG) potassium channel blockage by small molecules may cause severe cardiac side effects. Thus, it is crucial to screen compounds for activity on the hERG channels early in the drug discovery process. In this study, we collected 5299 hERG inhibitors with diverse chemical structures from a number of sources. Based on this dataset, we evaluated different machine learning (ML) and deep learning (DL) algorithms using various integer and binary type fingerprints. A training set of 3991 compounds was used to develop quantitative structure–activity relationship (QSAR) models. The performance of the developed models was evaluated using a test set of 998 compounds. Models were further validated using external set 1 (263 compounds) and external set 2 (47 compounds). Overall, models with integer type fingerprints showed better performance than models with no fingerprints, converted binary type fingerprints or original binary type fingerprints. Comparison of ML and DL algorithms revealed that integer type fingerprints are suitable for ML, whereas binary type fingerprints are suitable for DL. The outcomes of this study indicate that the rational selection of fingerprints is important for hERG blocker prediction.

## 1. Introduction

The human ether-a-go-go related gene (hERG) or KCNH2 gene encodes a voltage-gated potassium channel known as the hERG channel. This channel plays a key role in cardiac action potential repolarization. Reduced function of hERG causes potential action prolongation and increases the risk for potentially fatal ventricular arrhythmia, torsades de pointes. Therefore, preclinical hERG testing is essential in the drug discovery process to avoid cardiac toxicity [1]. Recently, many drugs such as astemizole, terfenadine, cisapride, thioridazine, grepafloxacin and sertindole were withdrawn from the market due to undesired cardiotoxicity effects [2,3]. Nowadays, the US Food and Drug Administration (FDA) demands in vitro hERG assay of lead compounds prior to clinical trials [4]. The side effects of unexpected hERG channel binding by drug candidates are a major challenge in the drug discovery process [5]. The development of an accurate prediction model for hERG channel blockers is crucial in the early stages of drug discovery and development.

Although the electron microscopy structure of the membrane protein hERG is known [6], its X-ray crystal structure is not available. Thus, structure-based hERG blocker prediction is challenging. However, a few structure-based hERG blocker predictions were attempted with homology modeling using structures of the related potassium ion channels as templates [7]. Several researchers have applied a ligand-based drug design approach to identify hERG blockers. They used various machine learning (ML) algorithms such as naïve Bayes (NB) [8], support vector machine (SVM) [9], random forest (RF) [10] and k-NN [11]. Sun et al. reported an NB model with a receiver operating characteristic (ROC) value of 0.87 on the basis of 1979 compounds [12]. Jia et al. applied SVM methods and atom-type descriptors without fingerprints on 1043 compounds to develop a model with accuracy of 0.94 [9]. Yap et al. developed a model with accuracy of 0.97 based on 310 compounds using similar methodology [13]. Marchese Robinson et al. built an RF model with Matthews correlation coefficient (MCC) of 0.83 using a dataset of 368 compounds. They used the extended-connectivity fingerprint (ECFP_4) for developing the model [14]. Kim et al. developed a model with accuracy of 0.96 using the RF algorithm on a dataset of 293 compounds [15]. Chavan et al. reported a k-NN model with accuracy of 0.55 on the basis of 1967 compounds [11]. The deep learning (DL) approach with artificial neural networks (ANN) has also been used to predict hERG blockers [16,17]. Cai et al. applied DL to 7889 compounds and obtained accuracy of 0.93 and an area under the receiver operating characteristic curve (AUC) value of 0.97 for the best model [17]. Zhang et al. applied DL to 1871 compounds and developed a model with accuracy of 0.78 [18]. Several research papers have reported the application of ML or DL techniques on hERG blockers. However, smaller datasets were used in the previous publications as compared to the recent papers utilizing the DL approach. Datasets for hERG blockers have grown in recent years.

Fingerprints used in the former ligand-based drug design studies on hERG as well as other targets were mostly binary types representing 0 or 1 [19,20,21,22]. Fingerprints describe chemical substructures as numerical category data. The number of a binary type fingerprint is usually restricted to 1024 bits [23]. The integer type fingerprints describe chemical structures in more detail with various substructures. They produce a large number of category data types useful for ML. Although integer type fingerprints indicate diversity of chemical substructures, they are limited to commercial software such as the Pipeline Pilot (PP) module of Discovery Studio (DS) software (BIOVIA, San Diego, CA, USA) [24]. In the present study, we calculated integer as well as binary type fingerprints for a dataset of hERG blockers. Integer type fingerprints were computed using PP and they included extended-connectivity fingerprints (ECFP_2, ECFP_4 and ECFP_6) and functional-class fingerprints (FCFP_2, FCFP_4 and FCFP_6). Binary type chemistry development kit (CDK) fingerprints (standard, extended and graph) were computed using the R package. We used both types of fingerprints for ML and DL. Our results show that rational selection of fingerprints is important for hERG blocker prediction. We have discussed the advantages and disadvantages of integer and binary type fingerprints in ML and DL.

## 2. Results

### 2.1. Dataset Splitting

**Figure 1:**
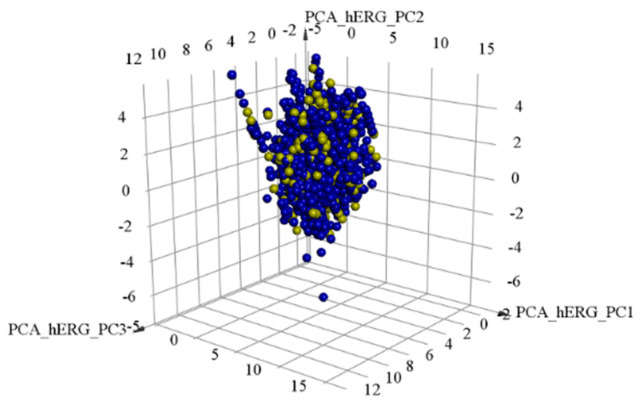
Principle component analysis (PCA) for the training and test sets of hERG blockers. Independent variables included partition coefficient (AlogP), molecular weight (MW), hydrogen-bond donor (HBD), hydrogen-bond acceptor (HBA), rotatable bond number (RBN), number of rings (Num Rings), number of aromatic rings (Num Arom Rings) and molecular fractional polar surface area (MFPSA). Blue and yellow spheres indicate training and test sets, respectively.

**Figure 2:**
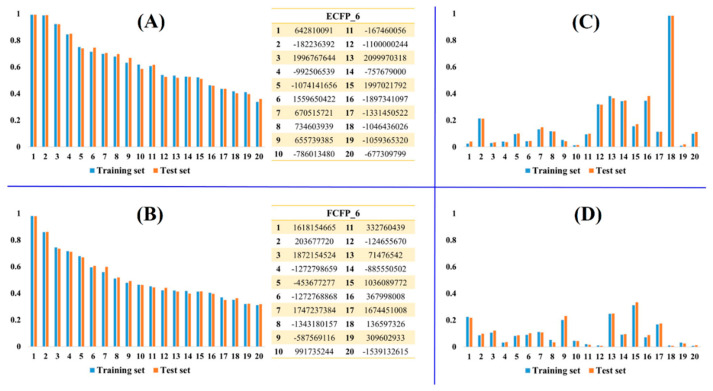
Fingerprint frequency of the training and test sets of hERG blockers. X-axis denotes fingerprint identifier whereas Y-axis denotes the fingerprint frequency. Representative integer type PP fingerprints: (**A**) ECFP_6 and (**B**) FCFP_6. Representative binary type chemistry development kit (CDK) fingerprints: (**C**) standard and (**D**) extended. For validating the appropriate split of the dataset into training and test sets, the top 20 of PP integer fingerprint identifiers were selected as representatives depending on the inclusion order. Furthermore, the top 20 of CDK binary fingerprint identifiers were chosen as representatives depending on the binary sequence (1024 bits). Fingerprint frequency was calculated as the observed number of unique fingerprint identifiers/the total number of chemical data (training set = 3991 and test set = 998).

As discussed in the methodology section, a dataset consisting of hERG blockers was divided into the training and test sets in the ratio 4:1. The training and test sets consisted of 3991 and 998 compounds, respectively. Principle component analysis (PCA) was performed to verify the diversity of the chemical space of the dataset. PCA analysis with eight common descriptors, including partition coefficient (AlogP), molecular weight (MW), hydrogen-bond donor (HBD), hydrogen-bond acceptor (HBA), rotatable bond number (RBN), number of rings (Num Rings), number of aromatic rings (Num Arom Rings) and molecular fractional polar surface area (MFPSA), showed high chemical diversity of the compounds within the training and test sets (Figure 1). Generally, PCA descriptors are indirect numeric type representations of chemical structures. However, fingerprints representing chemical substructures are not numeric type. They are Boolean type indicating existence or nonexistence (1 or 0) of a unique fingerprint. Therefore, additional integer and binary type fingerprints were computed to validate the division of the dataset. As shown in Figure 2, integer type PP fingerprints (ECFP_6 and FCFP_6) and binary type CDK fingerprints (standard and extended) showed similar fingerprint frequency for the training and test sets. Binary type CDK fingerprints displayed relatively lower fingerprint frequency than integer type PP fingerprints. This is due to the limited size of the binary type CDK fingerprints (1024 bits). PCA analysis and fingerprint frequency calculations validate the appropriate splitting of the dataset into training and test sets.

### 2.2. Model Building and Evaluation

**Figure 3:**
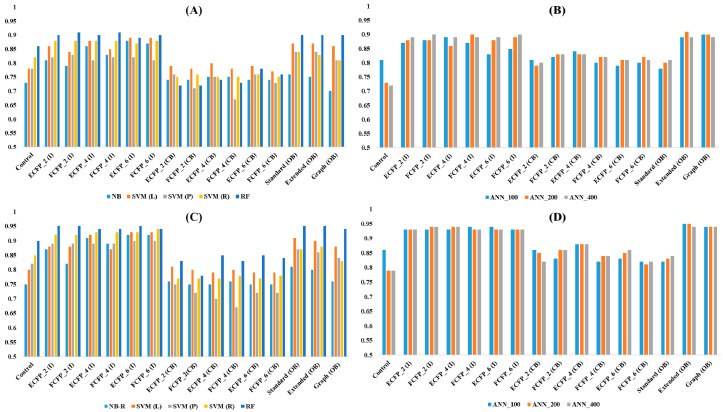
Models built using integer and binary type fingerprints for hERG blockers. X-axis denotes fingerprint types whereas Y-axis denotes the performance. Control lacking fingerprint only include descriptors, I: integer type PP fingerprints, CB: converted binary type PP fingerprints, OB: original binary type CDK fingerprints, NB: naïve Bayes, SVM: support vector machine (L: linear, P: polynomial, R: radial), RF: random forest, ANN: artificial neural network (layer size 100, 200, and 400) for deep learning. (**A**,**B**) Accuracy value for the models using machine learnings (**A**) and deep learning (**B**). (**C**,**D**) AUC value for machine learnings (**C**) and deep learning (**D**).

The training set comprising 3991 compounds was used to build the models and the test set consisting of 998 compounds was used to evaluate the performance of the developed models. Different integer and binary type fingerprints were utilized for model building (Figure 3 and Supplementary materials Tables S1 and S2). All control models which lacked fingerprints and included only descriptors (AlogP, MW, HBD, HBA, RBN, Num Rings, Num Arom Rings and MFPSA) as features showed average predictive accuracy (Q) of 0.77 (ML = 0.79 and DL = 0.75) and an average area under the receiver operating characteristic curve (AUC) value of 0.82 (ML = 0.82 and DL = 0.81). The addition of fingerprints in the features improved the performance of the models. All models including integer type PP fingerprints (ECFP_2, FCFP_2, ECFP_4, FCFP_4, ECFP_6 and FCFP_6) displayed average Q and AUC values of 0.87 (ML = 0.86 and DL = 0.88) and 0.92 (ML = 0.91 and DL = 0.93), respectively. Integer type fingerprints were converted to binary type using the “Convert Fingerprint” module of PP. The conversion of integer type to binary type fingerprints decreased the performance of the models. All models including converted binary type PP fingerprints demonstrated average Q and AUC values of 0.77 (ML = 0.75 and DL = 0.81) and 0.81 (ML = 0.77 and DL = 0.84), respectively. The open-source based CDK fingerprints (standard, extended and graph) are originally binary type and cannot be converted to integer type. Models with original binary type CDK fingerprints showed a comparatively higher performance than converted binary type PP fingerprints. All models, including binary type CDK fingerprints, exhibited average Q and AUC values of 0.86 (ML = 0.83 and DL = 0.89) and 0.90 (ML = 0.87 and DL = 0.93), respectively. Integer type PP fingerprints exhibited a higher performance than original binary type CDK fingerprints for ML. However, integer type PP fingerprints demonstrated a slightly lower performance than original binary type CDK fingerprints for DL. Compared to integer type PP fingerprints and original binary type CDK fingerprints, converted binary type PP fingerprints showed lower performance for both ML and DL. Comparison of different algorithms revealed that the RF algorithm produced better models than others. Models derived using the RF algorithm exhibited average Q and AUC values of 0.90 and 0.95, respectively, for both integer type PP fingerprints as well as original binary type CDK fingerprints. Furthermore, the RF algorithm produced the best model with FCFP_2 integer type PP fingerprint. This model showed Q and AUC values of 0.91 and 0.95, respectively.

### 2.3. External Validation

**Figure 4:**
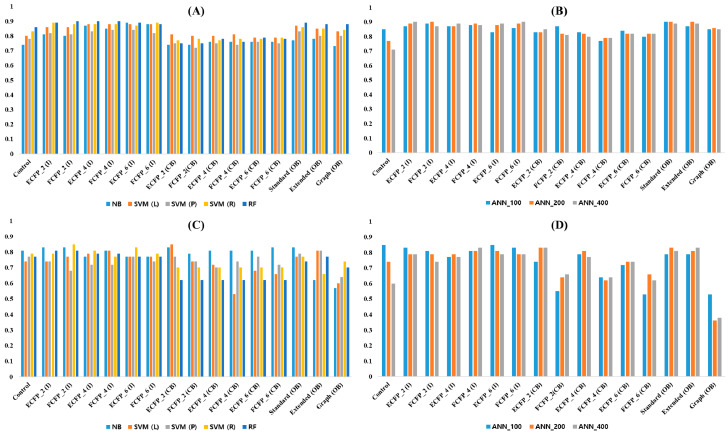
Model prediction to external sets. X-axis denotes fingerprint types whereas Y-axis denotes the performance for accuracy. Control lacking fingerprint include only descriptors, I: integer type PP fingerprints, CB: converted binary type PP fingerprints, OB: original binary type CDK fingerprints, NB: naïve Bayes, SVM: support vector machine (L: linear, P: polynomial, R: radial), RF: random forest, ANN: artificial neural network (layer size 100, 200, and 400) for deep learning. (**A**,**B**) Ex-1 prediction for machine learnings (**A**) and deep learning (**B**). (**C**,**D**) Ex-2 prediction for machine learnings (**C**) and deep learning (**D**).

Two external sets were used for further validation of the models. As discussed in the methodology section, external set 1 (Ex-1) from ChEMBL database and external set 2 (Ex-2) from recent publications [25,26,27,28,29] consisted of 263 and 47 hERG blockers, respectively. Model predictions for Ex-1 and Ex-2 using integer and binary type fingerprints are summarized in Figure 4 and Tables S3–S6 (Supplementary materials), respectively. The control models exhibited an average Q value of 0.79 (ML = 0.80 and DL = 0.78) for Ex-1. The Ex-1 showed average Q values of 0.87 (ML = 0.86 and DL = 0.88), 0.80 (ML = 0.77 and DL = 0.82) and 0.86 (ML = 0.83 and DL = 0.88) for integer type PP fingerprints, converted binary type PP fingerprints and original binary type CDK fingerprints, respectively. Ex-1 displayed higher predictive accuracy for both integer type PP fingerprints and original binary type CDK fingerprints than for the control models. However, integer type PP fingerprints produced comparatively higher predictive accuracy than original binary type CDK fingerprints. Converted binary type PP fingerprints showed predictive accuracy similar to the control models. Integer type PP fingerprints exhibited higher predictive accuracy than original binary type CDK fingerprints for ML. However, integer type PP fingerprints exhibited predictive accuracy similar to the original binary type CDK fingerprints for DL. Control models exhibited an average Q value of 0.76 (ML = 0.79 and DL = 0.73) for Ex-2. The Ex-2 showed average Q values of 0.80 (ML = 0.80 and DL = 0.80), 0.71 (ML = 0.71 and DL = 0.70) and 0.70 (ML = 0.72 and DL = 0.68) for integer type PP fingerprints, converted binary type PP fingerprints and original binary type CDK fingerprints, respectively. Compared to the control models, Ex-2 displayed higher predictive accuracy for integer type PP fingerprints while it showed lower predictive accuracy for original binary type CDK fingerprints and converted binary type PP fingerprints. Integer type PP fingerprints exhibited higher predictive accuracy for both ML and DL as compared to the original binary type CDK fingerprints and converted binary type PP fingerprints.

In accordance with the model-building results, converted binary type PP fingerprints reduced the predictive accuracy of the models. Original binary type CDK fingerprints exhibited comparatively higher predictive accuracy than converted binary type PP fingerprints for Ex-1. However, Ex-2 displayed slightly lower predictive accuracy for original binary type CDK fingerprints as compared to converted binary type PP fingerprints. This might be due to the small data size of the Ex-2 (47 compounds). Further parameters for external validation of the developed models are provided in Tables S3–S6 (Supplementary materials). These included true positive (TP), true negative (TN), false positive (FP), and false negative (FN). It can be seen in the supplementary tables that TN predictions were higher than FN. This might be because compounds in the whole dataset as well as the external sets were intended to develop as inhibitors for other drug targets but they also inhibited hERG, to produce toxicity. During model building, the RF algorithm displayed the same average Q value of 0.90 for both integer type PP fingerprints as well as original binary type CDK fingerprints. However, the RF model predictions for Ex-1 and Ex-2 using integer type PP fingerprints were found to be better than original binary type CDK fingerprints. The RF models with integer type PP fingerprints showed average Q values of 0.89 and 0.79 for Ex-1 and Ex-2, respectively. The RF models with original binary type CDK fingerprints exhibited average Q values of 0.88 and 0.74 for Ex-1 and Ex-2, respectively. The best model that was obtained using the RF algorithm and FCFP_2 integer type PP fingerprint showed Q values of 0.90 and 0.81 for Ex-1 and Ex-2, respectively.

## 3. Discussion

The unwanted blockage of the hERG channel by drug candidates could lead to fatal cardiotoxicity. Thus, it is essential to screen compounds for activity on hERG channels early in the drug discovery process to decrease the risk of a drug candidate failing in preclinical safety studies. Due to the unavailability of the crystal structure of the hERG channel, researchers mainly use the ligand-based drug design approach to identify hERG blockers. Several ML and DL approaches have been applied. However, fingerprints used in the majority of the previous studies were mostly binary type [30,31]. These fingerprints are limited in size and generally restricted to 1024 bits. The integer type fingerprints describe chemical structures in more detail, but they are limited to commercial software. Fingerprints of the integer type have not been explored much for the prediction of hERG blockers or for the prediction of other drug target inhibitors. In this study, we computed both integer and binary type fingerprints for a dataset of hERG blockers and evaluated various ML (NB, SVM, RF and Bagging) and DL (ANN) algorithms. A training set of 3991 compounds was used to develop QSAR models. The performance of the developed models was evaluated using a test set of 998 compounds. Models were further validated using two external sets (Ex-1: 263 compounds and Ex-2: 47 compounds).

The overall results showed that addition of fingerprints to the descriptors improved the performance of the models. Models with integer type PP fingerprints displayed slightly better performance than models with binary type CDK fingerprints. Conversion of integer type PP fingerprints to binary type PP fingerprints reduced the performance of the models. Except for small-sized Ex-2, models with binary type CDK fingerprints showed better performance than converted binary type PP fingerprints. Comparison of different algorithms revealed that models built by RF outperformed those built by other algorithms. This is in agreement with a previous study that reported high performance of the RF models [17]. The RF algorithm demonstrated a similar performance for both integer type PP fingerprints as well as original binary type CDK fingerprints during model building. However, the RF models with integer type PP fingerprints showed comparatively better predictions for both the external sets than the RF models with original binary type CDK fingerprints. The best model was obtained using the RF algorithm and FCFP_2 integer type PP fingerprint. Comparison of ML and DL algorithms revealed that ML models with integer type PP fingerprints demonstrated better performance and predictions than ML models with binary type CDK fingerprints. On the other hand, DL models performed slightly better with binary type CDK fingerprints as compared to DL models with integer type PP fingerprints. However, DL models exhibited similar predictions for both integer type PP fingerprints and binary type CDK fingerprints for Ex-1. In the case of small-sized Ex-2, ML and DL models showed better predictions with integer type PP fingerprints as compared to ML and DL models with binary type CDK fingerprints.

The outcomes of the study suggested that integer type fingerprints improved the performance and predictive ability of QSAR models. Although integer type fingerprints could be applied to both ML and DL, these fingerprints did not improve the predictive accuracy of DL models significantly. Moreover, they required long computation time due to the large number of features. Numerous fingerprint identifiers caused memory problems in some ANN packages such as “nnet”. Due to memory problems, integer type fingerprints needed to be converted to binary type for DL. Our results demonstrated that conversion of fingerprints from integer to binary type reduced the performance and the predictive ability of the models in both ML and DL. Compared to integer type fingerprints, original binary type fingerprints produced DL models with slightly better performance. Furthermore, the predictive ability of DL models with binary type fingerprints was comparable to DL models with integer type fingerprints. Accordingly, binary type fingerprints are recommended for DL. Binary type fingerprints were suitable for both ML and DL due to their limited size. However, binary type fingerprints produced ML models with comparatively lower performance and predictive ability than ML models with integer type fingerprints. Consequently, integer type fingerprints are recommended for ML. In conclusion, rational selection of fingerprints is important for hERG blocker prediction.

## 4. Materials and Methods

### 4.1. Dataset

A dataset consisting of 5252 compounds with hERG inhibition values (IC_50_ and K_i_) was obtained from the ChEMBL database [32]. The reported K_i_ values were converted into IC_50_ values. In accordance with previous studies, compounds with IC_50_ ≤ 1 μM and IC_50_ > 10 μM were classified as active and inactive compounds, respectively [15,16]. Active and inactive compounds were defined as 1 and 0, respectively. Prior to splitting the dataset into training and test sets, 263 compounds (5% of the dataset) were extracted by random selection as Ex-1 using DS 2019 software (BIOVIA, San Diego, CA, USA). The remaining dataset with 4989 compounds was randomly partitioned into training and test sets in the ratio 4:1. The training and test sets consisted of 3991 and 998 compounds, respectively. In addition to the ChEMBL dataset, 47 hERG blockers reported in recent research papers were collected as Ex-2 from recent publications [25,26,27,28,29]. Active and inactive compounds for the external set 2 (Ex-2) were defined in the same way as discussed for the ChEMBL dataset (Table 1). The training set was used for the model building whereas the test and external sets (Ex-1 and Ex-2) were used for model evaluation.

### 4.2. Fingerprint Calculation

The PP integer type fingerprints were computed using the “molecular fingerprint” module of PP. The “atom abstraction” option was set to atom type and functional class for calculating ECFP and FCFP fingerprints, respectively. The “maximum distance” option was set to 2, 4 and 6 for generating different types of ECFP and FCFP fingerprints (ECFP_2, FCFP_2, ECFP_4, FCFP_4, ECFP_6 and FCFP_6). Integer type fingerprints were converted to binary type fingerprints using the “Convert Fingerprint” module of PP. CDK binary type fingerprints (standard, extended and graph) were computed with “rcdk” package [33] of R (version 3.5.2, R Core Team, Vienna, Austria) [34]. The “get.fingerprint” function was used for the calculation of the fingerprints. The fingerprint frequency was determined for validating the appropriate split of the dataset into training and test sets. Fingerprint frequency was calculated as the observed number of unique fingerprint identifiers/the total number of chemical data (training set = 3991 and test set = 998).

### 4.3. Model Building

In this study, we evaluated one DL algorithm: ANN, and four ML algorithms: NB, SVM, RF and bagging. DS 2019 software was used for NB and bagging because it supports only these two algorithms. R package was employed for NB, SVM, RF, ANN. NB is available in both R and DS packages. NB-DS computes only integer type fingerprints but it is possible to calculate both integer as well as binary type fingerprints using NB-R. We utilized both the packages to evaluate NB. The “create Bayesian model” protocol of DS was used for NB-DS. The laplacian value was set to the default value of 1. The “create recursive partitioning model” protocol of DS was employed for decision-tree based bagging such as RF. The number of trees was set to 100. The “e1071” package [35] of R was utilized for NB and SVM. NB models were created using the “naïveBayes” function. The Laplace parameter was set to 1. SVM models were developed using the “svm” function. The linear, polynomial and radial methods were implemented for SVM. While implementing, linear, polynomial and radial kernel functions were selected. The “randomForest” package [36] of R was used for a decision-tree based RF. The number of trees was set to 100. The “h2o” package [37] of R was utilized for ANN with three hidden layers. The “Rectifier activation” function was employed, and the number of iterations was set to 20. The layer size parameter was set to 100, 200, and 400 for three hidden layers. Descriptors were calculated using DS. These included AlogP, MW, HBD, HBA, RBN, Num Rings, Num Arom Rings and MFPSA. Except for the control models, all developed models included fingerprints. Control models comprised only descriptors.

### 4.4. Model Evaluation

Model performance was evaluated in terms of predictive accuracy (Q), the area under the receiver operating characteristic curve (AUC), true positive (TP), true negative (TN), false positive (FP) and false negative (FN). The “calculate molecular property” protocol of DS 2019 software and “predict” function of R package were used for the model evaluation.

## Figures and Tables

**Table 1:** Dataset used in the present study.

Source	Set	Compounds	Active Compounds	Inactive Compounds
ChEMBL database (Total compounds: 5252)	Training set	3991	1201	2790
	Test set	998	278	720
	Ext-1	263	67	196
Recent research papers (Total compounds: 47)	Ext-2	47	18	29

## Supplementary Materials

The following are available online. Table S1. Models built using integer type fingerprints. Table S2. Models built using binary type fingerprints. Table S3. Model prediction for external set 1 using integer type fingerprints. Table S4. Model prediction for external set 1 using binary type fingerprints. Table S5. Model prediction for external set 2 using integer type fingerprints. Table S6. Model prediction for external set 2 using binary type fingerprints. The supplementary excel data for the compound list and detailed model prediction data used in this research for hERG blocker prediction.
