# Supplementary material for: The Study on the hERG Blocker Prediction Using Chemical Fingerprint Analysis
Source: Molecules. 2020 Jun 4;25(11):2615. doi: 10.3390/molecules25112615 (PMC7321128; doi:10.3390/molecules25112615)
Supplement: Supplementary file 1 [file molecules-25-02615-s001.zip › Supplementary_revised.pdf]

SUPPLEMENTARY DATA

# The Study on the hERG Blocker Prediction Using Chemical Fingerprint Analysis

Kwang-Eun Choi, Anand Balupuri, Nam Sook Kang [*]

*Graduate School of New Drug Discovery and Development, Chungnam National University, Daejeon 305-764, Republic of Korea*

**Table S1.** Models built using integer type fingerprints.

| Algorithm | Score | Control (no fingerprints) | PP (integer type FP) ECFP_2 | FCFP_2 | ECFP_4 | FCFP_4 | ECFP_6 | FCFP_6 |
|---|---|---|---|---|---|---|---|---|
| NB-DS | Q | 0.71 | 0.80 | 0.76 | 0.85 | 0.83 | 0.86 | 0.86 |
| | AUC | 0.79 | 0.88 | 0.84 | 0.91 | 0.90 | 0.92 | 0.92 |
| NB-R | Q | 0.73 | 0.81 | 0.79 | 0.86 | 0.83 | 0.88 | 0.87 |
| | AUC | 0.75 | 0.87 | 0.82 | 0.91 | 0.89 | 0.92 | 0.92 |
| SVM (linear) | Q | 0.78 | 0.86 | 0.84 | 0.88 | 0.85 | 0.89 | 0.89 |
| | AUC | 0.80 | 0.88 | 0.88 | 0.92 | 0.87 | 0.93 | 0.93 |
| SVM (polynomial) | Q | 0.78 | 0.82 | 0.83 | 0.81 | 0.82 | 0.82 | 0.81 |
| | AUC | 0.82 | 0.89 | 0.89 | 0.89 | 0.89 | 0.90 | 0.90 |
| SVM (radial) | Q | 0.82 | 0.88 | 0.88 | 0.88 | 0.88 | 0.87 | 0.88 |
| | AUC | 0.85 | 0.92 | 0.92 | 0.93 | 0.93 | 0.93 | 0.94 |
| RF (100 trees) | Q | 0.86 | 0.90 | 0.91 | 0.90 | 0.91 | 0.89 | 0.90 |
| | AUC | 0.90 | 0.95 | 0.95 | 0.94 | 0.94 | 0.95 | 0.94 |
| Bagging-DS | Q | 0.80 | 0.86 | 0.87 | 0.86 | 0.87 | 0.87 | 0.87 |

| | | | | | | | | |
|---|---|---|---|---|---|---|---|---|
| (100 trees) | AUC | 0.87 | 0.92 | 0.92 | 0.92 | 0.92 | 0.92 | 0.92 |
| ANN (100 size) | Q | 0.81 | 0.87 | 0.88 | 0.89 | 0.87 | 0.83 | 0.85 |
| | AUC | 0.86 | 0.93 | 0.93 | 0.93 | 0.94 | 0.94 | 0.93 |
| ANN (200 size) | Q | 0.73 | 0.88 | 0.88 | 0.86 | 0.90 | 0.88 | 0.89 |
| | AUC | 0.79 | 0.93 | 0.94 | 0.94 | 0.93 | 0.93 | 0.93 |
| ANN (400 size) | Q | 0.72 | 0.89 | 0.90 | 0.89 | 0.89 | 0.89 | 0.90 |
| | AUC | 0.79 | 0.93 | 0.94 | 0.94 | 0.93 | 0.93 | 0.93 |

Note- Q: predictive accuracy; AUC: area under the receiver operating characteristic curve.

**Table S2.** Models built using binary type fingerprints.

| Algorithm | Score | Control (no fingerprints) | Converted PP (binary) | | | | | | CDK (binary) | | |
|---|---|---|---|---|---|---|---|---|---|---|---|
| | | | ECFP_2 | FCFP_2 | ECFP_4 | FCFP_4 | ECFP_6 | FCFP_6 | Standard | Extended | Graph |
| NB-R | Q | 0.73 | 0.74 | 0.74 | 0.75 | 0.75 | 0.74 | 0.74 | 0.76 | 0.75 | 0.70 |
| | AUC | 0.75 | 0.76 | 0.75 | 0.75 | 0.76 | 0.75 | 0.75 | 0.81 | 0.8 | 0.76 |
| SVM (linear) | Q | 0.78 | 0.79 | 0.78 | 0.80 | 0.78 | 0.79 | 0.77 | 0.87 | 0.87 | 0.86 |
| | AUC | 0.80 | 0.81 | 0.80 | 0.79 | 0.80 | 0.79 | 0.79 | 0.91 | 0.90 | 0.88 |
| SVM (polynomial) | Q | 0.78 | 0.76 | 0.71 | 0.75 | 0.67 | 0.76 | 0.73 | 0.84 | 0.84 | 0.81 |
| | AUC | 0.82 | 0.75 | 0.72 | 0.70 | 0.67 | 0.72 | 0.72 | 0.87 | 0.86 | 0.84 |
| SVM (radial) | Q | 0.82 | 0.75 | 0.76 | 0.75 | 0.75 | 0.76 | 0.75 | 0.84 | 0.83 | 0.81 |
| | AUC | 0.85 | 0.77 | 0.77 | 0.77 | 0.78 | 0.77 | 0.78 | 0.87 | 0.88 | 0.83 |
| RF (100 trees) | Q | 0.86 | 0.72 | 0.72 | 0.74 | 0.73 | 0.78 | 0.76 | 0.90 | 0.90 | 0.90 |
| | AUC | 0.90 | 0.83 | 0.78 | 0.85 | 0.83 | 0.85 | 0.84 | 0.95 | 0.95 | 0.94 |
| ANN (100 size) | Q | 0.81 | 0.82 | 0.84 | 0.80 | 0.79 | 0.80 | 0.78 | 0.89 | 0.90 | 0.88 |
| | AUC | 0.86 | 0.83 | 0.88 | 0.82 | 0.83 | 0.82 | 0.82 | 0.95 | 0.94 | 0.92 |
| ANN (200 size) | Q | 0.73 | 0.83 | 0.83 | 0.82 | 0.81 | 0.82 | 0.80 | 0.91 | 0.90 | 0.88 |
| | AUC | 0.79 | 0.86 | 0.88 | 0.84 | 0.85 | 0.81 | 0.83 | 0.95 | 0.94 | 0.92 |
| ANN (400 size) | Q | 0.72 | 0.83 | 0.83 | 0.82 | 0.81 | 0.81 | 0.81 | 0.89 | 0.89 | 0.89 |
| | AUC | 0.79 | 0.86 | 0.88 | 0.84 | 0.86 | 0.82 | 0.84 | 0.94 | 0.94 | 0.92 |

Note- Q: predictive accuracy; AUC: area under the receiver operating characteristic curve.

**Table S3.** Model prediction for external set-1 using integer type fingerprints.

| Algorithm | Parameter | Control (No fingerprints) | PP (integer type FP) | | | | | |
|---|---|---|---|---|---|---|---|---|
| | | | ECFP_2 | FCFP_2 | ECFP_4 | FCFP_4 | ECFP_6 | FCFP_6 |
| NB-DS | Q | 0.69 | 0.80 | 0.73 | 0.86 | 0.83 | 0.86 | 0.88 |
| | TN | 134 | 162 | 147 | 174 | 167 | 173 | 178 |
| | FN | 20 | 18 | 22 | 14 | 16 | 15 | 14 |
| | FP | 62 | 34 | 49 | 22 | 29 | 23 | 18 |
| | TP | 47 | 49 | 45 | 53 | 51 | 52 | 53 |
| NB-R | Q | 0.74 | 0.81 | 0.80 | 0.87 | 0.85 | 0.89 | 0.88 |
| | TN | 166 | 166 | 166 | 179 | 172 | 183 | 181 |
| | FN | 38 | 19 | 23 | 16 | 16 | 17 | 17 |
| | FP | 30 | 30 | 30 | 17 | 24 | 13 | 15 |
| | TP | 29 | 48 | 44 | 51 | 51 | 50 | 50 |
| SVM (linear) | Q | 0.80 | 0.86 | 0.86 | 0.88 | 0.88 | 0.88 | 0.88 |
| | TN | 184 | 186 | 185 | 185 | 184 | 184 | 185 |
| | FN | 40 | 28 | 25 | 20 | 20 | 20 | 20 |
| | FP | 12 | 10 | 11 | 11 | 12 | 12 | 11 |
| | TP | 27 | 39 | 42 | 47 | 47 | 47 | 47 |
| SVM (polynomial) | Q | 0.78 | 0.82 | 0.81 | 0.83 | 0.84 | 0.84 | 0.82 |
| | TN | 192 | 186 | 185 | 180 | 183 | 184 | 182 |
| | FN | 54 | 37 | 38 | 28 | 29 | 31 | 33 |
| | FP | 4 | 10 | 11 | 16 | 13 | 12 | 14 |
| | TP | 13 | 30 | 29 | 39 | 38 | 36 | 34 |
| SVM (radial) | Q | 0.83 | 0.89 | 0.88 | 0.88 | 0.88 | 0.87 | 0.89 |
| | TN | 181 | 189 | 185 | 184 | 188 | 179 | 185 |
| | FN | 30 | 22 | 20 | 20 | 23 | 16 | 18 |
| | FP | 15 | 7 | 11 | 12 | 8 | 17 | 11 |
| | TP | 37 | 45 | 47 | 47 | 44 | 51 | 49 |
| RF | Q | 0.86 | 0.89 | 0.90 | 0.90 | 0.90 | 0.89 | 0.88 |

| | | | | | | | | |
|---|---|---|---|---|---|---|---|---|
| (100 trees) | TN | 186 | 194 | 194 | 193 | 193 | 192 | 190 |
| | FN | 27 | 27 | 25 | 24 | 24 | 26 | 25 |
| | FP | 10 | 2 | 2 | 3 | 3 | 4 | 6 |
| | TP | 40 | 40 | 42 | 43 | 43 | 41 | 42 |
| Bagging-DS (100 trees) | Q | 0.80 | 0.83 | 0.86 | 0.83 | 0.86 | 0.83 | 0.87 |
| | TN | 165 | 172 | 178 | 172 | 177 | 172 | 180 |
| | FN | 21 | 21 | 20 | 20 | 19 | 20 | 18 |
| | FP | 31 | 24 | 18 | 24 | 19 | 24 | 16 |
| | TP | 46 | 46 | 47 | 47 | 48 | 47 | 49 |
| ANN (100 size) | Q | 0.85 | 0.87 | 0.89 | 0.87 | 0.88 | 0.83 | 0.86 |
| | TN | 178 | 179 | 182 | 183 | 180 | 159 | 170 |
| | FN | 21 | 18 | 15 | 21 | 16 | 9 | 12 |
| | FP | 18 | 17 | 14 | 13 | 16 | 37 | 26 |
| | TP | 46 | 49 | 52 | 46 | 51 | 58 | 55 |
| ANN (200 size) | Q | 0.77 | 0.89 | 0.90 | 0.87 | 0.89 | 0.88 | 0.89 |
| | TN | 154 | 183 | 185 | 174 | 187 | 182 | 180 |
| | FN | 18 | 15 | 14 | 12 | 21 | 17 | 14 |
| | FP | 42 | 13 | 11 | 22 | 9 | 14 | 16 |
| | TP | 49 | 52 | 53 | 55 | 46 | 50 | 53 |
| ANN (400 size) | Q | 0.71 | 0.90 | 0.87 | 0.89 | 0.88 | 0.89 | 0.90 |
| | TN | 138 | 186 | 178 | 183 | 179 | 187 | 186 |
| | FN | 17 | 17 | 16 | 17 | 14 | 21 | 15 |
| | FP | 58 | 10 | 18 | 13 | 17 | 9 | 10 |
| | TP | 50 | 50 | 51 | 50 | 53 | 46 | 52 |

Note- Q: predictive accuracy; TP: true positive; TN: true negative; FP: false positive; FN: false negative.

**Table S4.** Model prediction for external set-1 using binary type fingerprints.

| Algorithm | Parameter | Control (No fingerprints) | Converted PP (binary) | | | | | | CDK (binary) | | |
|---|---|---|---|---|---|---|---|---|---|---|---|
| | | | ECFP_2 | FCFP_2 | ECFP_4 | FCFP_4 | ECFP_6 | FCFP_6 | Standard | Extended | Graph |
| NB-R | Q | 0.74 | 0.74 | 0.74 | 0.76 | 0.76 | 0.76 | 0.76 | 0.77 | 0.78 | 0.73 |
| | TN | 166 | 161 | 166 | 166 | 169 | 168 | 168 | 159 | 160 | 148 |
| | FN | 38 | 34 | 39 | 33 | 35 | 34 | 35 | 24 | 22 | 23 |
| | FP | 30 | 35 | 30 | 30 | 27 | 28 | 28 | 37 | 36 | 48 |
| | TP | 29 | 33 | 28 | 34 | 32 | 33 | 32 | 43 | 45 | 44 |
| SVM (linear) | Q | 0.80 | 0.81 | 0.80 | 0.80 | 0.81 | 0.79 | 0.79 | 0.87 | 0.85 | 0.83 |
| | TN | 184 | 182 | 182 | 180 | 182 | 182 | 181 | 183 | 179 | 179 |
| | FN | 40 | 37 | 38 | 36 | 37 | 40 | 40 | 20 | 23 | 28 |
| | FP | 12 | 14 | 14 | 16 | 14 | 14 | 15 | 13 | 17 | 17 |
| | TP | 27 | 30 | 29 | 31 | 30 | 27 | 27 | 47 | 44 | 39 |
| SVM (polynomial) | Q | 0.78 | 0.75 | 0.72 | 0.75 | 0.74 | 0.76 | 0.75 | 0.83 | 0.80 | 0.80 |
| | TN | 192 | 184 | 187 | 185 | 162 | 191 | 196 | 178 | 175 | 189 |
| | FN | 54 | 54 | 64 | 54 | 35 | 57 | 65 | 28 | 32 | 45 |
| | FP | 4 | 12 | 9 | 11 | 34 | 5 | 0 | 18 | 21 | 7 |
| | TP | 13 | 13 | 3 | 13 | 32 | 10 | 2 | 39 | 35 | 22 |
| SVM (radial) | Q | 0.83 | 0.77 | 0.78 | 0.77 | 0.78 | 0.78 | 0.79 | 0.86 | 0.85 | 0.84 |
| | TN | 181 | 180 | 181 | 179 | 181 | 179 | 181 | 184 | 182 | 183 |
| | FN | 30 | 45 | 43 | 44 | 42 | 41 | 41 | 26 | 25 | 30 |
| | FP | 15 | 16 | 15 | 17 | 15 | 17 | 15 | 12 | 14 | 13 |
| | TP | 37 | 22 | 24 | 23 | 25 | 26 | 26 | 41 | 42 | 37 |
| RF (100 trees) | Q | 0.86 | 0.75 | 0.75 | 0.78 | 0.76 | 0.79 | 0.78 | 0.89 | 0.88 | 0.88 |
| | TN | 186 | 196 | 196 | 196 | 196 | 196 | 196 | 191 | 189 | 191 |
| | FN | 27 | 67 | 67 | 59 | 63 | 54 | 59 | 25 | 24 | 27 |

| | | | | | | | | | | | |
|---|---|---|---|---|---|---|---|---|---|---|---|
| | FP | 10 | 0 | 0 | 0 | 0 | 0 | 0 | 5 | 7 | 5 |
| | TP | 40 | 0 | 0 | 8 | 4 | 13 | 8 | 42 | 43 | 40 |
| ANN (100 size) | Q | 0.85 | 0.83 | 0.87 | 0.83 | 0.77 | 0.84 | 0.80 | 0.90 | 0.87 | 0.85 |
| | TN | 178 | 178 | 184 | 180 | 169 | 186 | 176 | 187 | 182 | 174 |
| | FN | 21 | 27 | 21 | 30 | 33 | 31 | 33 | 16 | 19 | 18 |
| | FP | 18 | 18 | 12 | 16 | 27 | 10 | 20 | 9 | 14 | 22 |
| | TP | 46 | 40 | 46 | 37 | 34 | 36 | 34 | 51 | 48 | 49 |
| ANN (200 size) | Q | 0.77 | 0.83 | 0.82 | 0.82 | 0.79 | 0.82 | 0.82 | 0.90 | 0.90 | 0.86 |
| | TN | 154 | 182 | 170 | 179 | 172 | 186 | 176 | 191 | 189 | 183 |
| | FN | 18 | 30 | 22 | 31 | 32 | 38 | 27 | 20 | 20 | 24 |
| | FP | 42 | 14 | 26 | 17 | 24 | 10 | 20 | 5 | 7 | 13 |
| | TP | 49 | 37 | 45 | 36 | 35 | 29 | 40 | 47 | 47 | 43 |
| ANN (400 size) | Q | 0.71 | 0.85 | 0.81 | 0.80 | 0.79 | 0.82 | 0.82 | 0.89 | 0.89 | 0.85 |
| | TN | 138 | 181 | 168 | 172 | 172 | 181 | 181 | 184 | 183 | 180 |
| | FN | 17 | 25 | 23 | 28 | 31 | 32 | 33 | 18 | 17 | 23 |
| | FP | 58 | 15 | 28 | 24 | 24 | 15 | 15 | 12 | 13 | 16 |
| | TP | 50 | 42 | 44 | 39 | 36 | 35 | 34 | 49 | 50 | 44 |

Note- Q: predictive accuracy; TP: true positive; TN: true negative; FP: false positive; FN: false negative.

**Table S5.** Model prediction for external set-2 using integer type fingerprints.

| Algorithm | Parameter | Control (no fingerprints) | PP (integer type FP) | | | | | |
|---|---|---|---|---|---|---|---|---|
| | | | ECFP_2 | FCFP_2 | ECFP_4 | FCFP_4 | ECFP_6 | FCFP_6 |
| NB-DS | Q | 0.85 | 0.85 | 0.85 | 0.79 | 0.81 | 0.77 | 0.79 |
| | TN | 28 | 29 | 29 | 29 | 29 | 29 | 29 |
| | FN | 6 | 7 | 7 | 10 | 9 | 11 | 10 |
| | FP | 1 | 0 | 0 | 0 | 0 | 0 | 0 |
| | TP | 12 | 11 | 11 | 8 | 9 | 7 | 8 |
| NB-R | Q | 0.81 | 0.83 | 0.83 | 0.77 | 0.81 | 0.77 | 0.77 |
| | TN | 29 | 29 | 29 | 29 | 29 | 29 | 29 |
| | FN | 9 | 8 | 8 | 11 | 9 | 11 | 11 |
| | FP | 0 | 0 | 0 | 0 | 0 | 0 | 0 |
| | TP | 9 | 10 | 10 | 7 | 9 | 7 | 7 |
| SVM (linear) | Q | 0.74 | 0.74 | 0.77 | 0.79 | 0.81 | 0.77 | 0.77 |
| | TN | 26 | 26 | 28 | 29 | 29 | 29 | 29 |
| | FN | 9 | 9 | 10 | 10 | 9 | 11 | 11 |
| | FP | 3 | 3 | 1 | 0 | 0 | 0 | 0 |
| | TP | 9 | 9 | 8 | 8 | 9 | 7 | 7 |
| SVM (polynomial) | Q | 0.77 | 0.74 | 0.68 | 0.72 | 0.72 | 0.77 | 0.74 |
| | TN | 29 | 28 | 29 | 29 | 29 | 29 | 29 |
| | FN | 11 | 11 | 15 | 13 | 13 | 11 | 12 |
| | FP | 0 | 1 | 0 | 0 | 0 | 0 | 0 |
| | TP | 7 | 7 | 3 | 5 | 5 | 7 | 6 |
| SVM (radial) | Q | 0.79 | 0.79 | 0.85 | 0.81 | 0.77 | 0.83 | 0.79 |
| | TN | 29 | 29 | 29 | 29 | 29 | 29 | 29 |
| | FN | 10 | 10 | 7 | 9 | 11 | 8 | 10 |
| | FP | 0 | 0 | 0 | 0 | 0 | 0 | 0 |
| | TP | 8 | 8 | 11 | 9 | 7 | 10 | 8 |
| RF (100 trees) | Q | 0.77 | 0.81 | 0.81 | 0.79 | 0.79 | 0.77 | 0.77 |
| | TN | 29 | 29 | 29 | 29 | 29 | 29 | 29 |
| | FN | 11 | 9 | 9 | 10 | 10 | 11 | 11 |

| | | | | | | | | |
|---|---|---|---|---|---|---|---|---|
| | FP | 0 | 0 | 0 | 0 | 0 | 0 | 0 |
| | TP | 7 | 9 | 9 | 8 | 8 | 7 | 7 |
| Bagging-DS (100 trees) | Q | 0.83 | 0.89 | 0.83 | 0.89 | 0.87 | 0.87 | 0.87 |
| | TN | 28 | 29 | 29 | 29 | 29 | 29 | 29 |
| | FN | 7 | 5 | 8 | 5 | 6 | 6 | 6 |
| | FP | 1 | 0 | 0 | 0 | 0 | 0 | 0 |
| | TP | 11 | 13 | 10 | 13 | 12 | 12 | 12 |
| ANN (100 size) | Q | 0.85 | 0.83 | 0.81 | 0.77 | 0.81 | 0.85 | 0.83 |
| | TN | 29 | 29 | 29 | 29 | 29 | 24 | 27 |
| | FN | 7 | 8 | 9 | 11 | 9 | 2 | 6 |
| | FP | 0 | 0 | 0 | 0 | 0 | 5 | 2 |
| | TP | 11 | 10 | 9 | 7 | 9 | 16 | 12 |
| ANN (200 size) | Q | 0.74 | 0.79 | 0.79 | 0.79 | 0.81 | 0.81 | 0.79 |
| | TN | 23 | 28 | 29 | 28 | 29 | 29 | 29 |
| | FN | 6 | 9 | 10 | 9 | 9 | 9 | 10 |
| | FP | 6 | 1 | 0 | 1 | 0 | 0 | 0 |
| | TP | 12 | 9 | 8 | 9 | 9 | 9 | 8 |
| ANN (400 size) | Q | 0.60 | 0.79 | 0.74 | 0.77 | 0.83 | 0.79 | 0.79 |
| | TN | 16 | 29 | 28 | 29 | 29 | 29 | 29 |
| | FN | 6 | 10 | 11 | 11 | 8 | 10 | 10 |
| | FP | 13 | 0 | 1 | 0 | 0 | 0 | 0 |
| | TP | 12 | 8 | 7 | 7 | 10 | 8 | 8 |

Note- Q: predictive accuracy; TP: true positive; TN: true negative; FP: false positive; FN: false negative.

**Table S6.** Model prediction for external set-2 using binary type fingerprints.

| Algorithm | Parameter | Control (no fingerprints) | Converted PP (binary) | | | | | | CDK (binary) | | |
|---|---|---|---|---|---|---|---|---|---|---|---|
| | | | ECFP_2 | FCFP_2 | ECFP_4 | FCFP_4 | ECFP_6 | FCFP_6 | Standard | Extended | Graph |
| NB-R | Q | 0.81 | 0.83 | 0.79 | 0.81 | 0.81 | 0.81 | 0.83 | 0.83 | 0.62 | 0.57 |
| | TN | 29 | 29 | 27 | 29 | 28 | 29 | 29 | 29 | 19 | 17 |
| | FN | 9 | 8 | 8 | 9 | 8 | 9 | 8 | 8 | 8 | 8 |
| | FP | 0 | 0 | 2 | 0 | 1 | 0 | 0 | 0 | 10 | 12 |
| | TP | 9 | 10 | 10 | 9 | 10 | 9 | 10 | 10 | 10 | 10 |
| SVM (linear) | Q | 0.74 | 0.85 | 0.74 | 0.72 | 0.53 | 0.68 | 0.66 | 0.77 | 0.81 | 0.60 |
| | TN | 26 | 29 | 24 | 25 | 15 | 23 | 20 | 28 | 29 | 17 |
| | FN | 9 | 7 | 7 | 9 | 8 | 9 | 7 | 10 | 9 | 7 |
| | FP | 3 | 0 | 5 | 4 | 14 | 6 | 9 | 1 | 0 | 12 |
| | TP | 9 | 11 | 11 | 9 | 10 | 9 | 11 | 8 | 9 | 11 |
| SVM (polynomial) | Q | 0.77 | 0.77 | 0.74 | 0.70 | 0.74 | 0.77 | 0.72 | 0.79 | 0.81 | 0.64 |
| | TN | 29 | 29 | 28 | 28 | 27 | 29 | 29 | 29 | 29 | 24 |
| | FN | 11 | 11 | 11 | 13 | 10 | 11 | 13 | 10 | 9 | 12 |
| | FP | 0 | 0 | 1 | 1 | 2 | 0 | 0 | 0 | 0 | 5 |
| | TP | 7 | 7 | 7 | 5 | 8 | 7 | 5 | 8 | 9 | 6 |
| SVM (radial) | Q | 0.79 | 0.70 | 0.70 | 0.70 | 0.70 | 0.70 | 0.70 | 0.77 | 0.66 | 0.74 |
| | TN | 29 | 28 | 28 | 28 | 28 | 28 | 28 | 27 | 22 | 26 |
| | FN | 10 | 13 | 13 | 13 | 13 | 13 | 13 | 9 | 9 | 9 |
| | FP | 0 | 1 | 1 | 1 | 1 | 1 | 1 | 2 | 7 | 3 |
| | TP | 8 | 5 | 5 | 5 | 5 | 5 | 5 | 9 | 9 | 9 |
| RF (100 trees) | Q | 0.77 | 0.62 | 0.62 | 0.62 | 0.62 | 0.62 | 0.62 | 0.74 | 0.77 | 0.70 |
| | TN | 29 | 29 | 29 | 29 | 29 | 29 | 29 | 29 | 29 | 29 |

| | | | | | | | | | | | |
|---|---|---|---|---|---|---|---|---|---|---|---|
| | FN | 11 | 18 | 18 | 18 | 18 | 18 | 18 | 12 | 11 | 14 |
| | FP | 0 | 0 | 0 | 0 | 0 | 0 | 0 | 0 | 0 | 0 |
| | TP | 7 | 0 | 0 | 0 | 0 | 0 | 0 | 6 | 7 | 4 |
| ANN (100 size) | Q | 0.85 | 0.74 | 0.55 | 0.79 | 0.64 | 0.72 | 0.53 | 0.79 | 0.79 | 0.53 |
| | TN | 29 | 25 | 18 | 28 | 18 | 26 | 16 | 28 | 28 | 13 |
| | FN | 7 | 8 | 10 | 9 | 6 | 10 | 9 | 9 | 9 | 6 |
| | FP | 0 | 4 | 11 | 1 | 11 | 3 | 13 | 1 | 1 | 16 |
| | TP | 11 | 10 | 8 | 9 | 12 | 8 | 9 | 9 | 9 | 12 |
| ANN (200 size) | Q | 0.74 | 0.83 | 0.64 | 0.81 | 0.62 | 0.74 | 0.66 | 0.83 | 0.81 | 0.36 |
| | TN | 23 | 27 | 20 | 28 | 20 | 27 | 21 | 29 | 29 | 8 |
| | FN | 6 | 6 | 8 | 8 | 9 | 10 | 8 | 8 | 9 | 9 |
| | FP | 6 | 2 | 9 | 1 | 9 | 2 | 8 | 0 | 0 | 21 |
| | TP | 12 | 12 | 10 | 10 | 9 | 8 | 10 | 10 | 9 | 9 |
| ANN (400 size) | Q | 0.60 | 0.83 | 0.66 | 0.77 | 0.64 | 0.74 | 0.62 | 0.81 | 0.83 | 0.38 |
| | TN | 16 | 26 | 20 | 27 | 20 | 24 | 18 | 29 | 28 | 8 |
| | FN | 6 | 5 | 7 | 9 | 8 | 7 | 7 | 9 | 7 | 8 |
| | FP | 13 | 3 | 9 | 2 | 9 | 5 | 11 | 0 | 1 | 21 |
| | TP | 12 | 13 | 11 | 9 | 10 | 11 | 11 | 9 | 11 | 10 |

Note- Q: predictive accuracy; TP: true positive; TN: true negative; FP: false positive; FN: false negative.
